# Molecular Dynamics Studies on the Conformational Transitions of Adenylate Kinase: A Computational Evidence for the Conformational Selection Mechanism

**DOI:** 10.1155/2013/628536

**Published:** 2013-06-27

**Authors:** Jie Ping, Pei Hao, Yi-Xue Li, Jing-Fang Wang

**Affiliations:** ^1^Pathogen Diagnostic Center, Institut Pasteur of Shanghai Chinese Academy of Sciences, Shanghai 200025, China; ^2^Shanghai Center for Bioinformation Technology, 100 Qinzhou Road, Shanghai 200235, China; ^3^Bioinformatics Center, Key Laboratory of Systems Biology, Shanghai Institutes for Biological Sciences, Chinese Academy of Sciences, Shanghai 200031, China; ^4^Key Laboratory of Systems Biomedicine (Ministry of Education), Shanghai Center for Systems Biomedicine, Shanghai Jiao Tong University, Shanghai 200240, China

## Abstract

*Escherichia coli* adenylate kinase (ADK) is a monomeric phosphotransferase enzyme that catalyzes reversible transfer of phosphoryl group from ATP to AMP with a large-scale domain motion. The detailed mechanism for this conformational transition remains unknown. In the current study, we performed long time-scale molecular dynamics simulations on both open and closed states of ADK. Based on the structural analyses of the simulation trajectories, we detected over 20 times conformational transitions between the open and closed states of ADK and identified two novel conformations as intermediate states in the catalytic processes. With these findings, we proposed a possible mechanism for the large-scale domain motion of *Escherichia coli* ADK and its catalytic process: (1) the substrate free ADK adopted an open conformation; (2) ATP bound with LID domain closure; (3) AMP bound with NMP domain closure; (4) phosphoryl transfer occurred with ATP, and AMP converted into two ADPs, and no conformational transition was detected in the enzyme; (5) LID domain opened with one ADP released; (6) another ADP released with NMP domain open. As both open and closed states sampled a wide range of conformation transitions, our simulation strongly supported the conformational selection mechanism for *Escherichia coli* ADK.

## 1. Introduction

**Figure 1:**
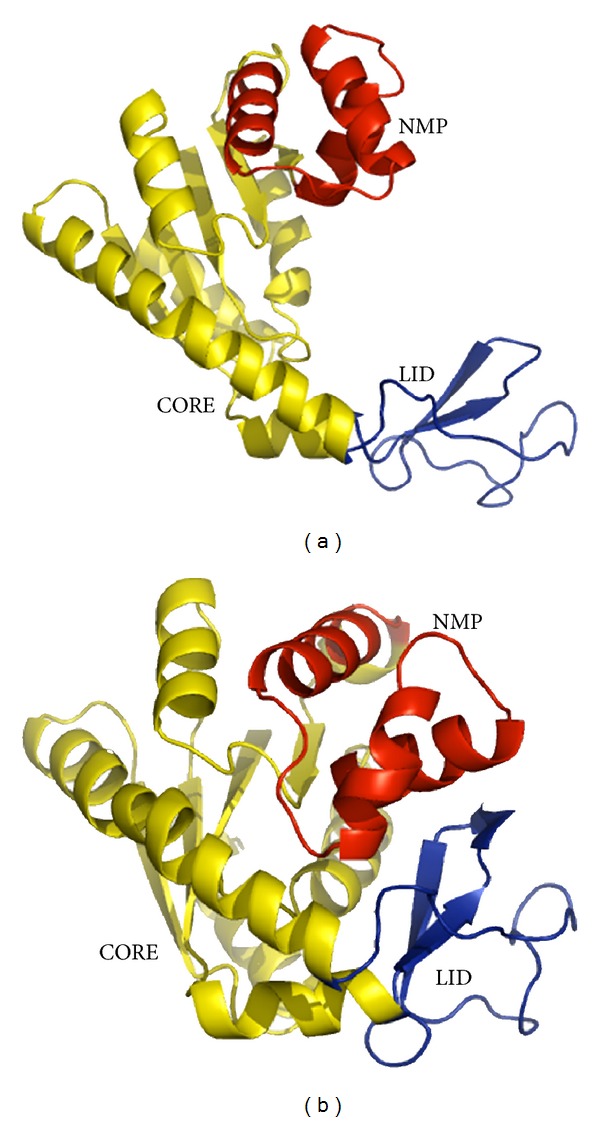
The three-dimensional structures of *Escherichia coli* adenylate kinase in the open (a) and closed (b) conformations. The crystal structures 4ake.pdb and 1ake.pdb are selected as the open and closed state of ADK, respectively. NMP (residues 30–67), CORE (residues 1–29, 68–117, and 161–214), and LID (residues 118–167) domains are colored in red, yellow, and blue, respectively.

*Escherichia coli* adenylate kinase (ADK) is a monomeric phosphotransferase enzyme regulating energy homeostasis in cells by catalyzing reversible transfer of a phosphoryl group from ATP to AMP. The structure of ADK is well studied in the past few years, and by now more than 20 crystal structures of ADK from *Escherichia coli* and other organisms in the absence and presence of substrates have been released in the protein structure databases. According to these crystal studies, the structure of ADK is mainly composed of three major components (Figure 1): a core domain (residues 1–29, 68–117, and 161–214), an AMP binding lid domain (also called NMP domain, residues 30–67), as well as an ATP binding lid domain (also called LID domain, residues 118–167). These basic components are shared by many other kinases and ATPases. As characterized by structural [1–4], biophysical [5, 6], and computational studies [7–10], ADK is believed to adopt an open conformation in the absence of substrates (Figure 1(a)), and with ATP or AMP binding the LID and NMP domains of this enzyme undergo large conformational transitions, leading to a closed conformation (Figure 1(b)). However, during the large conformational transitions, the core domain is comparatively rigid with the structural motions mainly located on the LID and NMP domains. This large conformational transition, especially opening of the nucleotide binding lids required for the catalysis and product release, occur on the microsecond-millisecond times scale [11].

Large-scale conformational alterations are thought to mediate allosteric regulation, which are related to the protein function in signal transduction, immune response, and enzymatic activity. Thus, a fundamental problem is to understand the mechanism for the large-scale conformational transitions. To answer this question, two models are proposed: the induced-fit model and the population-shift model. In the induced-fit model, substrate binding is believed to induce conformational alterations in the active site to cause a new conformation for the entire enzyme. Contrarily, in the population-shift model, the enzyme is thought to adopt a conformational equilibrium among many native states, and substrates are able to selectively bind to a suitable native conformation, shifting the equilibrium toward the binding conformation.

Several computational works have been done in recent years to study the conformational transitions of ADK. All atomic molecular dynamics simulations have been successfully used to reveal the structural trajectories of ADK during the large conformational transitions [7–9, 12–21]. However, these studies still remain on the nanosecond times scale much smaller than the times scale on which the large conformational transitions of ADK occur. Additionally, coarse-grained simulations [22–26], normal mode analysis [27–29], and plastic/elastic network approaches [30–33] are also employed to generate transition pathways of ADK. However, these theoretical studies are controversial for adopting the simple harmonic potential approximation instead of all-atom models, which is less convincing due to the intermediate structures far away from the native states. To this end, we performed long time-scale molecular dynamics simulations on both open and closed state of ADK to study the large conformational transitions. 

## 2. Materials and Methods

### 2.1. Initial Structures

 Owing to playing an important role in cellular energy homeostasis, many good attempts have been made to study the three-dimensional (3D) structure of ADK. By now, 29 crystal structures of ADK and its homolog, in the absence and presence of substrates, are available in the protein structure databases [34–45]. In this study, the crystal structures (PDB ID: 4AKE [2] and 1AKE [46]) were selected as the initial structures for the further molecular dynamics simulations. The former was released in 1996 with 2.2 Å resolutions and treated as an open state of ADK in the current case. The latter was obtained in the presence of Ap_5_A with 1.9 Å resolutions and considered as a closed state of ADK in this study. Except for the polar hydrogen and heavy atoms of the enzyme and Ap_5_A, all other atoms including nonpolar hydrogen in both crystal structures were removed. The pKa values for each residue in ADK were calculated by Delphi [47, 48] as a Poisson-Boltzmann solver with a dielectric constant of 4. Hydrogen atoms were subsequently added to the enzyme with t-Leap procedure of Amber 11 package [49] based on the computational pKa values in the last step to give a total charge of −4. Then, the enzyme (together with Ap_5_A in the closed structure) was solvated in a simulation box with explicit TIP3P water molecules. To neutralize the system, 4 sodium ions were added to random place 4 water molecules in the simulation box. The atoms of the enzyme were parameterized by Amber force field parameters [50], while Ap_5_A was done by Antechamber module [51] in Amber 11 package.

### 2.2. Molecular Dynamics Simulations

Two simulation systems were involved in this study, with the crystal structures (PDB ID: 4AKE and 1AKE) as initial structures for the open and closed conformations, respectively. Both systems were subjected to the steepest descent energy minimization (~5000 steps) followed by conjugate gradient energy minimization for the next 5000 steps and subsequently equilibrated with the enzyme atoms (or ligand in the closed system) fixed for a short-time molecular dynamics simulations at 300 K to reduce the van der Waals conflicts. Finally, 10-nanosecond (ns) molecular dynamics simulations were performed for both systems under a constant temperature (300 K) by Amber 11 package [49] with periodic boundary conditions and NPT ensemble. Tweenty five frames were randomly selected from the first 10 ns simulation trajectories for both systems, and 10 independent molecular dynamics simulations (10 ns) were launched for each frame. For the simulations that were detected to have large scale conformational transitions, 200 ns molecular dynamics simulations were added using a GPU-accelerated approach. SHAKE algorithm with a tolerance of 10^−6^ was applied to constrain all bonds in both simulation systems [52, 53], and atom velocities for start-up runs were obtained according to the Maxwell distribution at 300 K [54, 55]. The isothermal compressibility was set to 4.5 × 10^−5^/bar for solvent simulations [56, 57]. The electrostatic interactions were treated by the particle mesh Ewald (PME) algorithm with interpolation order of 4 and a grid spacing of 0.12 nanometers (nm) [58, 59]. The van der Waals interactions were calculated by using a cut-off of 12 Å. All the molecular dynamics simulations were performed with a time step of 2 femtoseconds (fs), and coordinates for both systems were saved every 1 picosecond (ps).

## 3. Results and Discussion

### 3.1. Conformational Transitions

**Figure 2:**
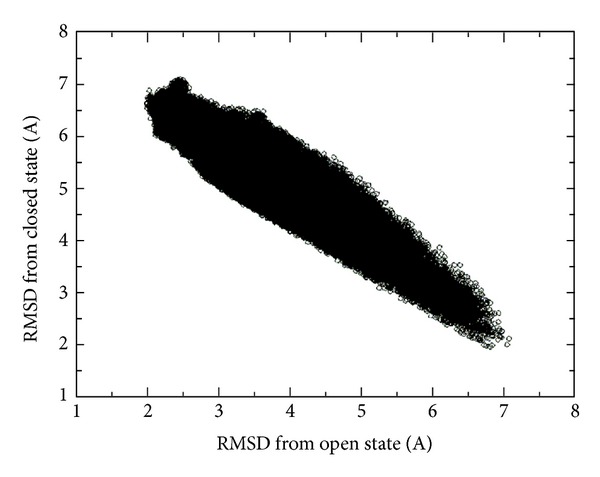
The RMS deviations of C*α* atoms from both open and closed conformation along the molecular dynamics trajectories. The open (4ake.pdb) and closed (1ake.pdb) conformations of ADK were specially labeled. The ribbon RMS deviation distribution indicated that large-scale conformational transitions were detected during our molecular dynamics simulations.

 As ADK has been well studied, a lot of kinetic and thermodynamic experimental data were released [60–64], based on which the large-scale conformational transitions for the NMP and LID domain opening are considered to be rate limiting for the catalytic reactions of the enzyme. However, it is really a challenging task to capture large-scale and long-time conformational transitions of proteins or enzymes for both experimental and computational methods. To check whether our simulations involved the large-scale conformational transitions of ADK, we calculated the RMS deviations of the C*α* atoms from both open (4ake.pdb) and closed (1ake.pdb) conformations of ADK along all the molecular dynamics simulation trajectories. As shown in Figure 2, the RMS deviation employed a ribbon distribution, indicating that the large-scale conformational transitions occurred in our molecular dynamics simulations. If no conformational transitions occurred, the 2D-RMS deviations should be divided into two major parts. Additionally, the 2D-RMS deviations located in the middle of the ribbon distribution might stand for the intermediate states. As reported by Shapiro and Meirovitch [64], the rate for ADK domain motions was about 52 ns. While we employed a series of simulations with a time scale of 200 ns, almost 4 times larger than the measured rates for ADK domain motions, thus it was expected that the conformational transitions should be detected in our molecular dynamics simulations.

**Figure 3:**
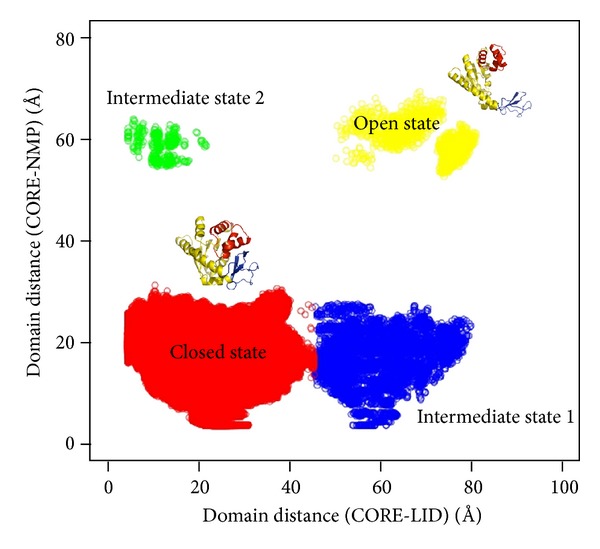
The mass center distances of NMP and LID domain with LID domain. The geometric center distances of NMP and CORE domains in the open and closed conformations were 62.7 and 18.4 Å, while those of LID and CORE domain for the open and closed conformations were 70.1 and 21.0 Å, respectively.

To confirm the conformational transitions for *Escherichia coli* ADK, we also measured the domain motion of ADK using the geometric center distances of NMP and LID domains with CORE domain along the molecular dynamics simulation trajectories. In the crystal structures, the geometric center distances of NMP and CORE domains in the open (4ake.pdb) and closed (1ake.pdb) conformations were 62.7 Å and 18.4 Å, while those for LID and CORE domain were 70.1 Å (open state) and 21.0 Å (closed state), respectively. As shown in Figure 3, the domain distances were clustered into four major parts. The ones colored in yellow and red, respectively, represented the open and closed conformations defined by the crystal structures, whereas the ones colored in blue and green were identified as novel configurations from the open and closed states. The blue ones in Figure 3 employed an open LID domain with a closed NMP domain, while the green ones had an open NMP domain with a closed LID domain. We believed that the blue and green parts in Figure 3 might be the intermediate states of ADK domain motions. 

### 3.2. Conformational Transition Pathway

**Figure 4:**
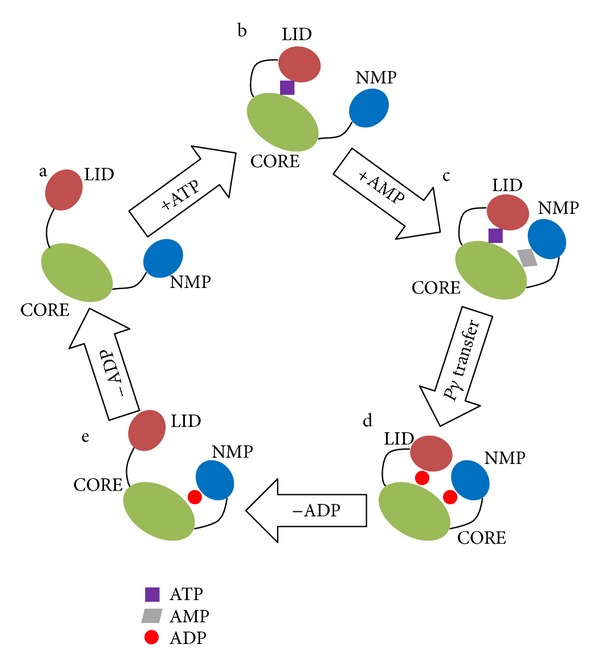
Conformational transition pathway and proposed catalytic mechanism of ADK. Model a, substrate free ADK with an open conformation. Model b, ATP bound form of ADK with a closed LID domain. Model c, ATP and AMP bound form of ADK with a closed conformation. Model d, two ADP bound forms of ADK with a closed conformation. Model e, one ADP bound form of ADK with a closed NMP domain.

 The large-scale domain motions were detected in the catalytic conversion of Mg^2+^–ATP + AMP → Mg^2+^–ADP + ADP by *Escherichia coli* ADK [65, 66]. Thus, the possible conformational transition pathway was thought to be associated with the catalytic mechanism for *Escherichia coli* ADK. According to the previous theoretical studies, LID domain motion was believed to precede NMP domain motion [6, 26, 33, 66]. One possible reason was that there was a stable salt bridge D118-K136 between LID and CORE, which could connect both domains by the strong contributions to the total enthalpic interactions. This salt bridge, also detected in several adenylate kinase structures of different species, was thought to have stabilizing function for the open conformation [9]. Another possible reason was that ATP binding in LID domain was found to have ability to assist NMP domain motion [66]. Based on these points, we proposed a possible conformational transition pathway and catalytic mechanism for *Escherichia coli* ADK (Figure 4). The substrate free enzyme adopted an open state. After ATP binding, ADK would close its LID domain first to form a novel configuration (model b in Figure 4 and intermediate state 2 in Figure 3). The closure motion of LID domain allowed AMP to bind in NMP domain binding site, resulting in the NMP domain closure (model c in Figure 4). With ATP and AMP binding, the enzyme would adopt a complete closed conformation. After phosphoryl transfer, ATP and AMP were converted into two ADP molecules. In this step, no conformational transition occurred, and thus the enzyme remained in its closed state (model d in Figure 4). When an ADP molecule was released from the ATP binding site, LID domain altered its closed configuration into an open state (model e in Figure 4 and intermediate state 1 in Figure 3). Subsequently, NMP domain opened via the bending of *α*2 helix toward *α*4 helix of CORE domain by nearly 15 degrees with respect to *α*3 helix, and the enzyme would adopt a complete open conformation (model a in Figure 4). This opening process of NMP domain (also AMP binding cleft) was considered to be involved in facilitating an efficient release of the formed product after catalysis.

Our proposed mechanism was also supported by some experimental results. Firstly, the complete closed conformation of ADK was found to be the only product-forming state [24]. In other words, to achieve its biological functions, ADK had to convert the open state (substrate free state) into the closed state. Secondly, The ATP binding site in LID domain was able to accommodate ATP, ADP, as well as AMP. ATP employed the highest binding affinity, whereas AMP had the lowest binding affinity [26]. Thirdly, the AMP binding site in NMP domain could only accommodate ADP and AMP [26]. Finally, the AMP binding site could only accommodate AMP in the intermediate state 2 with a closed LID domain and an open NMP domain [18]. Along the aforementioned pathway, a secondary structure analysis of the enzyme structure was performed using DSSP package to check the structural stability during the large-scale conformational transitions. As expected, no significant alterations in secondary structures were detected in CORE and NMP domain during the large-scale conformational transitions. For LID domain, some residues were found to bend and turn with few alterations in secondary structures. Thus, it was believed that ADK maintained its integrate structure with minute changes in its secondary structures, indicating that this enzyme behaved as a rigid body with flexible domains, and pathway detected in our study was reasonable.

Furthermore, our proposed mechanism was also in good agreement with previous theoretical studies. In 2008, Lu and Wang developed a coarse grained two-well model to study the conformational dynamics of ADK in microscopic detail [19]. They identified the LID-closing and NMP-closing pathways, providing theoretical evidence to our model (especially the transitions between model a and b and between model b and c). In 2008, Kubitzki and de Groot used TEE-REX molecular dynamics to study the conformational transitions of ADK and detected a pathway for the open-to-closed transitions [9]. Although a complete transition was not observed, the pathway they found was in accord with the transitions from model a to model c. In 2010, Prof. Head-Gordon and her co-workers used normal modes analyses to study the transition pathway [27]. Although a complete transition pathway was identified, they did not provide any explanation for the catalytic mechanism of ADK. Besides the aforementioned studies, many other works were involved in the free energy calculations during the conformational transitions [19–22], giving an indication that there were intermediate states during the conformational transitions.

### 3.3. Conformational Transition Mechanism

For the conformational transitions in proteins or enzymes, two different substrate binding mechanisms, induced-fit model, and conformational selection model can be proposed. The former is a model for enzyme-substrate interactions to describe that only the proper substrate is capable of inducing the conformational changes in the binding site, allowing the enzyme to perform its catalytic function [67]. The latter believes that the proteins or enzymes exist in the multiple conformations in the vicinity of its native state, and the ligand selectively binds to an active conformation to shift the equilibrium toward the binding conformation [68, 69]. In the current case, long time-scale molecular dynamics simulations were performed on *Escherichia coli* ADK, which can sample a large set of conformations between the open and closed states. Our computational results strongly supported the conformational selection model proposed for ADK [5, 70–74].

## 4. Conclusion

In conclusion, to study the large-scale domain motions in *Escherichia coli* ADK, we performed long time-scale molecular dynamics on both open and closed states of ADK. The two-dimensional RMS deviations of the C*α* atoms from both open and closed conformations along simulation trajectories confirmed the fact that conformational transitions between the open and closed conformation occurred during our simulations. Additionally, two significant intermediate states were identified by monitoring the domain distances between LID/NMP and CORE domains, one of which adopted an open LID domain with a closed NMP domain, and the other one employed an open NMP domain with a closed LID domain. Based on these computational results, we proposed a possible mechanism for the large scale conformational transitions and the catalytic function (Figure 4). The proposed mechanism was in good accordance with the previous experimental and theoretical studies, providing strong support to the conformational selection mechanism for *Escherichia coli* ADK.
